# A Medical Image Segmentation Method Based on Improved UNet 3+ Network

**DOI:** 10.3390/diagnostics13030576

**Published:** 2023-02-03

**Authors:** Yang Xu, Shike Hou, Xiangyu Wang, Duo Li, Lu Lu

**Affiliations:** 1 Institute of Disaster and Emergency Medicine, Tianjin University, Tianjin 300072, China; 2 Academy of Medical Engineering and Translational Medicine, Tianjin University, Tianjin 300072, China

**Keywords:** medical image segmentation, deep learning, UNet network, multi-scale skip connections, attention mechanism

## Abstract

In recent years, segmentation details and computing efficiency have become more important in medical image segmentation for clinical applications. In deep learning, UNet based on a convolutional neural network is one of the most commonly used models. UNet 3+ was designed as a modified UNet by adopting the architecture of full-scale skip connections. However, full-scale feature fusion can result in excessively redundant computations. This study aimed to reduce the network parameters of UNet 3+ while further improving the feature extraction capability. First, to eliminate redundancy and improve computational efficiency, we prune the full-scale skip connections of UNet 3+. In addition, we use the attention module called Convolutional Block Attention Module (CBAM) to capture more essential features and thus improve the feature expression capabilities. The performance of the proposed model was validated by three different types of datasets: skin cancer segmentation, breast cancer segmentation, and lung segmentation. The parameters are reduced by about 36% and 18% compared to UNet and UNet 3+, respectively. The results show that the proposed method not only outperformed the comparison models in a variety of evaluation metrics but also achieved more accurate segmentation results. The proposed models have lower network parameters that enhance feature extraction and improve segmentation performance efficiently. Furthermore, the models have great potential for application in medical imaging computer-aided diagnosis.

## 1. Introduction

Medical imaging can provide a wealth of information to help clinicians make the best diagnosis possible. However, current medical imaging diagnosis primarily relies on manual interpretation, which will add to doctors’ workload and lead to misjudgment operations. Computer-aided diagnosis has shown to be a reliable tool for reducing the strain on clinicians and shortening the time it takes to evaluate medical images [1,2,3]. Among these, the automatic segmentation of medical images is one of the current research hotspots.

Since deep learning has first been used, many researchers have employed convolutional neural networks (CNNs) to enable the automatic segmentation of medical images [4,5,6]. Representative CNNs models include FCN [7], SegNet [8], PSPNet [9], DeepLab [10,11], and UNet [12]. The encoder-decoder based UNet architecture, in particular, is widely used for medical image segmentation. It uses skip connections to combine the encoder’s low-level feature maps with the corresponding decoder’s high-level feature maps. However, the direct skip connections in UNet limit its capacity to capture abundant features [13]. UNet++ [14,15] further introduces nested and dense skip pathways in these connections, superimposing high-resolution features in the encoder layer to the deeper decoder layer, reducing the semantic gap between feature mappings. However, UNet++ has more parameters than UNet. In addition, the edge and location information of the image is easily diluted by down-sampling or up-sampling operations of the deep network, which does not make full use of the low-level feature maps. Subsequently, UNet 3+ [16] further aggregated feature maps from the full-scale perspective. Although UNet 3+ can capture the full-scale coarse-grained and fine-grained semantic feature maps, the features of adjacent layers contribute very similarly to the segmentation results when connected to the corresponding decoder layers. Therefore, full-scale feature fusion would have excessive redundant computations. By pruning the skip connections of UNet 3+ to reduce redundant computations, we proposed an optimized multi-scale skip connections segmentation architecture named Ref-UNet 3+. In addition, we also show that the segmentation performance of this model does not degrade.

In addition, many medical image segmentation and classification tasks have used the attention mechanism. It can make the neural network focus on and select significant features while suppressing unnecessary ones. Recent studies by many scholars are as follows: He et al. [17] proposed a novel classification model using the category attention block to identify diabetic retinopathy with small lesions and imbalanced data distribution. Hu et al. [18] proposed a parallel deep learning segmentation algorithm based on a hybrid attention mechanism that can extract multi-scale feature maps. Xiao et al. [19] proposed an MRI brain disease detection model based on transferred residual networks combined with a convolutional block attention module (CBAM), which has an excellent performance in two-class and multi-class tasks. Canayaz [20] proposed an EfficientNet consisting of attention blocks and proved that the attention mechanism plays a critical role in extracting deep-level features. Niu et al. [21] proposed a multi-scale attention-based convolutional neural network, enhancing the network’s feature expression ability. This study further introduces a lightweight attention mechanism module to the decoder [22], which is used to enhance the feature extraction and expression capabilities of the network.

In summary, the main contributions of this paper are as follows:

(1) We proposed an improved model Ref-UNet 3+, which reduced unnecessary redundant calculations.
(2) We used the current advanced attention module (CBAM) to enhance the feature extraction ability of the network.
(3) Algorithm comparison and quantitative analysis were performed on three different modalities of medical imaging, including skin cancer segmentation, breast cancer segmentation, and lung segmentation, demonstrating the better performance of our proposed model.

## 2. Methods

**Figure 1:**
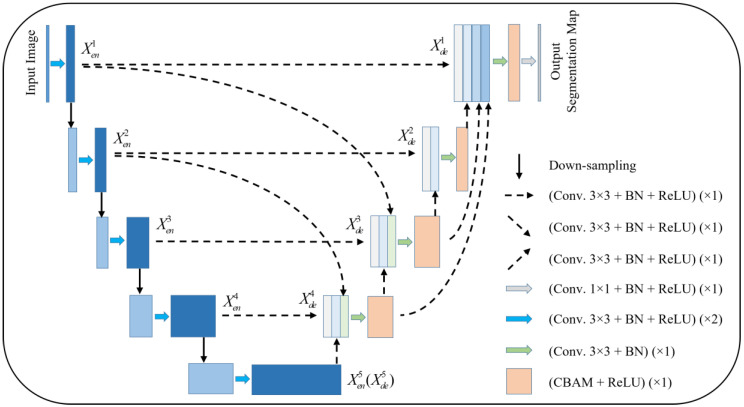
An improved UNet 3+ model combined with CBAM.

Figure 1 gives the proposed network model. The model is pruned on the basis of UNet 3+, and subsequently, an attention mechanism is added to the decoder layer. Taking $X_{de}^{3}$ as an example, the features from $X_{en}^{1}$, $X_{en}^{3}$, and $X_{de}^{4}$ are concatenated and sent to the CBAM module after a 3 × 3 convolution and BN layer, and subsequently sent to the next layer to complete the subsequent operations.

Compared with UNet and UNet 3+, the improved model provides more accurate segmentation effects with fewer parameters using multi-scale feature fusion and attention mechanisms.

### 2.1. Redesigned Multi-Scale Skip Connections

The redesigned skip connections consider the positive impact of specific multi-scale feature maps on segmentation. In UNet 3+, each decoder layer will have adjacent multi-scale feature maps with similar contributions to segmentation, resulting in excessive redundant computation. As an example, the feature map $X_{de}^{3}$ is obtained by $X_{en}^{1}$ and $X_{en}^{2}$ through different max pooling operations, respectively, the same-scale encoder layer $X_{en}^{3}$ is fed into a plain 3 × 3 convolution layer followed by a sigmoid function, and the larger-scale decoder layer $X_{de}^{5}$ and $X_{de}^{4}$ are fed into a 3 × 3 convolution layer followed by a bilinear up-sampling and a sigmoid function. However, the feature map $X_{en}^{2}$ and $X_{en}^{3}$, and the feature map $X_{de}^{5}$ and $X_{de}^{4}$ have little difference in their contribution to segmentation. As a result, our proposed model retains the low-level and high-level semantic feature information with an enormous contribution by pruning UNet 3+ accordingly.

Formally, we formulate the multi-scale skip connections: let i represent the current encoder-decoder layer and N refers to the total number of layers in the network. The stack of feature maps represented by $X_{de}^{i}$ is computed as:

(1) $$X_{de}^{i}=\left\{\begin{array}{cc} A(C([S(X_{en}^{i}),U{\left(X_{de}^{k}\right)}_{k=i+1}^{N-2}])), & i=1\\ A(C([S(X_{en}^{i}),U{\left(X_{de}^{k}\right)}_{k=i+1},M{\left(X_{en}^{j}\right)}_{j=i-2}])), & i>1,j>0 \end{array}\right.$$

where function $A\left(\cdot \right)$ represents the convolutional block attention module (CBAM) followed by a ReLU activation function. $C\left(\cdot \right)$ indicates a set of convolution and batch normalization operations. $S\left(\cdot \right)$ represents a convolution followed by batch normalization and a ReLU activation function. $U\left(\cdot \right)$ and $M\left(\cdot \right)$ denote up-sampling and down-sampling, respectively. $\left[\cdot \right]$ indicates the multi-scale feature maps concatenation layer.

### 2.2. CBAM in the Decoder

**Figure 2:**
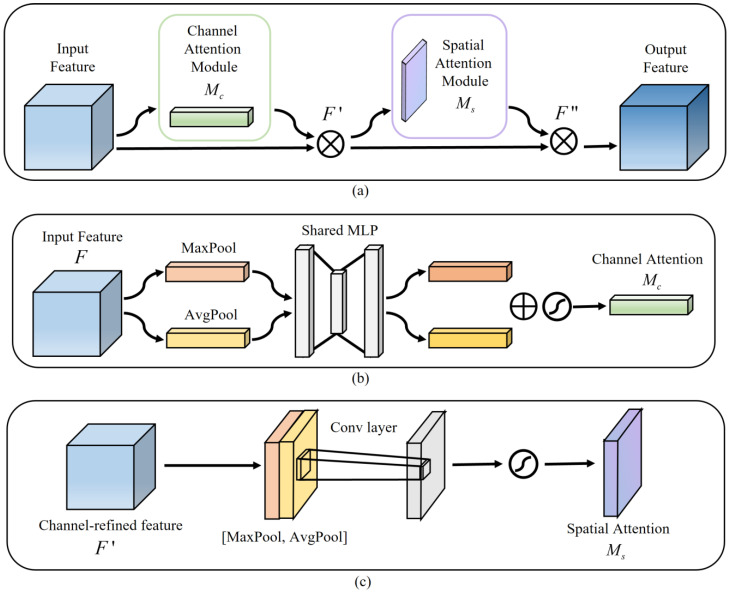
CBAM structure. (**a**) Convolutional Block Attention Module. (**b**) Channel Attention Module. (**c**) Spatial Attention Module.

Among the many attention models, CBAM is a lightweight feedforward convolutional neural network attention module that can be integrated into any CNN architecture for end-to-end training [22,23]. Figure 2a illustrates the CBAM structure, Figure 2b shows the channel attention, and Figure 2c indicates the spatial attention. A CBAM with channel attention and spatial attention facilitates the combination of more expressive feature information, thereby leading to a more efficient extraction of contextual information from images of various scales [24]. In our model, each decoder layer gets the feature map $F\in R^{C\times H\times W}$ fed into a convolution operation, and then this feature map F is considered the input feature map of CBAM. Secondly, the channel attention map $M_{C}\in R^{C\times 1\times 1}$ and channel-refined feature map $F^{\prime }$ are calculated, then the spatial attention map $M_{s}\in R^{1\times H\times W}$ is derived. Finally, the final feature map $F^{\prime \prime }$ is the output. The relevant calculation formulas are summarized as follows:

(2) $$F\prime =M_{c}(F)⊗F$$

(3) $$F\prime \prime =M_{s}(F\prime )⊗F\prime$$

where ⊗ represents the element-wise multiplication corresponding to the feature matrix.

## 3. Experiments and Results

**Figure 3:**
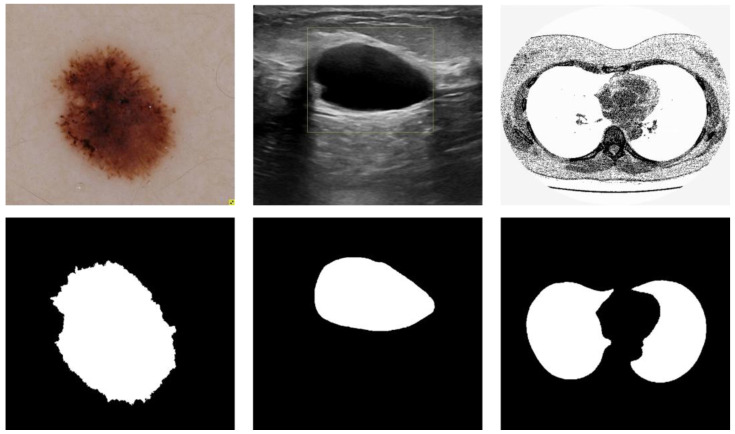
Medical image segmentation: skin cancer lesion segmentation on the left, breast cancer segmentation, and lung segmentation on the right.

We conducted experiments on three different types of medical imaging datasets to validate the performance of the proposed model. Figure 3 depicts three data samples. The models involved in this paper are implemented based on the deep learning frameworks Pytorch, and the experimental program language is Python. The experiments were run on a dual-core Intel(R) i7-11700 CPU with 32 GB of RAM and an NVIDIA GEFORCE RTX 3080 GPU with 10 GB of RAM. The running platform is Windows 10. The training parameters of the comparison models were kept consistent for the sake of fairness and rationality in the experiment, and the average of five random validation outcomes is the experimental result.

### 3.1. Datasets

#### 3.1.1. Skin Cancer Segmentation

Dataset used in this study was from the ISIC Challenge competition in 2018 [25], which is provided by https://www.kaggle.com/datasets/tschandl/isic2018-challenge-task1-data-segmentation/ (accessed on 15 November 2022). It consists of 2594 images and 2594 corresponding ground truth response masks. In this implementation, each sample was rescaled to 256 × 256 pixels.

#### 3.1.2. Breast Cancer Segmentation

The data reviews the medical images of breast cancer using ultrasound scans [26], which is provided by https://www.kaggle.com/datasets/aryashah2k/breast-ultrasound-images-dataset/ (accessed on 15 November 2022). The dataset consists of 780 images with an average image size of 500 × 500 pixels. The experimental data are 647 benign and malignant samples, which are resized to 256 × 256 pixels in this implementation.

#### 3.1.3. Lung Segmentation

The dataset is taken from The Lung Nodule Analysis (LUNA) and The Kaggle Data Science Bowl competition in 2017, which is provided by https://www.kaggle.com/datasets/kmader/finding-lungs-in-ct-data/ (accessed on 15 November 2022). The original image size was 512 × 512. In this implementation, each sample was rescaled to 256 × 256 pixels.

### 3.2. Quantitative Analysis Approaches

To evaluate the model reasonably, we considered the following evaluation indicators: accuracy (ACC), sensitivity (SE), precision (PRE), F1-score, Jaccard similarity (JS), and Dice coefficient (DC).

(4) $$\mathrm{ACC}=\frac{\mathrm{TP}+\mathrm{TN}}{\mathrm{TP}+\mathrm{TN}+\mathrm{FP}+\mathrm{FN}}$$

(5) $$\mathrm{SE}=\frac{\mathrm{TP}}{\mathrm{TP}+\mathrm{FN}}$$

(6) $$\mathrm{PRE}=\frac{\mathrm{TP}}{\mathrm{TP}+\mathrm{FP}}$$

(7) $$F1-\mathrm{score}=\frac{2*\mathrm{Precison}*\mathrm{Sensitivity}}{\mathrm{Precison}+\mathrm{Sensitivity}}$$

(8) $$\mathrm{JS}=\frac{\left|\mathrm{GT}\cap \mathrm{SR}\right|}{\left|\mathrm{GT}\cup \mathrm{SR}\right|}$$

(9) $$\mathrm{DC}=\frac{2*|\mathrm{GT}\cap \mathrm{SR}|}{\left|\mathrm{GT}|+|\mathrm{SR}\right|}$$

In Equations (4)–(6), True Positive (TP) represents the number of pixels correctly segmented by the target, False Positive (FP) represents the number of pixels incorrectly segmented by the background as the target, True Negative (TN) represents the number of pixels correctly segmented by the background, and False Negative (FN) represents the number of pixels incorrectly segmented by the target as the background. In Equations (8) and (9), Ground Truth (GT) and Segmentation Result (SR) denote the true labels and the generated prediction maps, respectively.

### 3.3. Loss Function

The model in this paper is an end-to-end deep learning network. The Dice coefficient loss is usually used as the loss function in medical image segmentation. The Dice coefficient is an ensemble similarity measure function to calculate the similarity of two samples and takes values in the range [0, 1].

The Dice coefficient loss is computed as:

(10) $$L_{dice}=1-\frac{2*|\mathrm{GT}\cap \mathrm{SR}|}{\left|\mathrm{GT}|+|\mathrm{SR}\right|}$$

where $\left|\mathrm{GT}\cap \mathrm{SR}\right|$ is the intersection between the label and the prediction. $\left|\mathrm{GT}\right|+\left|\mathrm{SR}\right|$ denotes the sum of the elements of the label and the prediction.

### 3.4. Results

#### 3.4.1. Skin Cancer Segmentation

**Figure 4:**
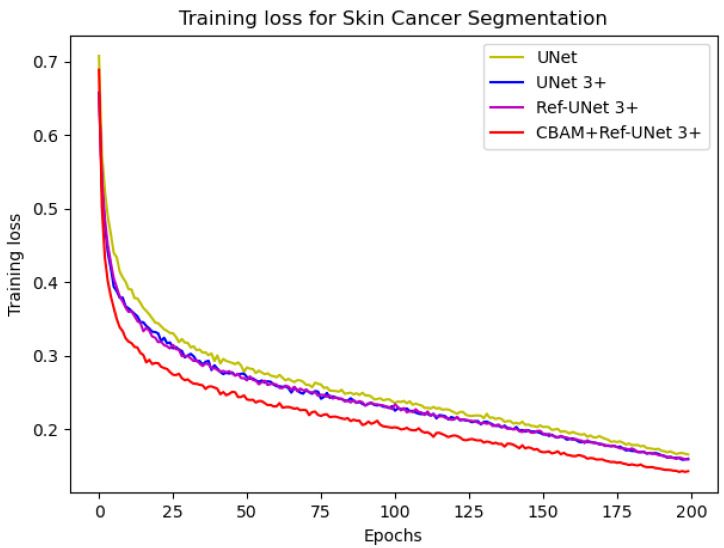
Comparison of training loss of different models for skin cancer segmentation.

**Figure 5:**
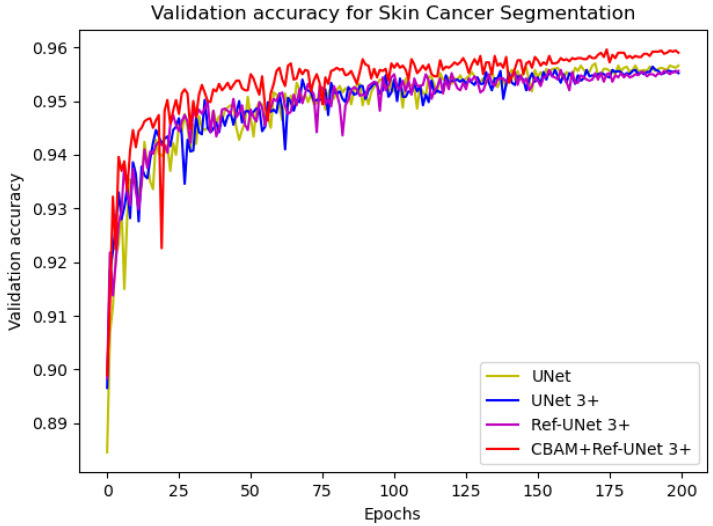
Comparison of validation accuracy of different models for skin cancer segmentation.

In this implementation, we adopted the ADAM [27] optimization technique with a weight decay of 0.0001. In addition, the data augmentation ratio was 0.5 and the learning rate was 2 × 10^−4^. The number of iterations was 200, and the loss function was dice coefficient loss. The encoder layer architecture of the U-shape network is 64—>128—>256—>512—>1024, and the decoder layers make corresponding adjustments according to different models. The proposed models Ref-UNet 3+ and CBAM+Ref-UNet 3+ were compared with UNet and UNet 3+ in terms of training loss and validation accuracy. The results are shown in Figure 4 and Figure 5. The proposed approach can be seen to achieve smaller loss, quicker convergence, and higher accuracy. This displays the suggested model’s remarkable robustness in a very straightforward way.

Table 1 shows the results of the 5-fold cross-validation and average. Our proposed models have excellent segmentation performance, with the number of parameters reduced roughly to 36% and 18%, compared to UNet and UNet 3+. Among them, CBAM+Ref-UNet 3+ surpasses UNet, UNet3+, and Ref-UNet 3+. The average F1-score in the testing phase achieved 0.8970, which is 0.76 and 1.00 points higher than UNet and UNet 3+, respectively. In addition, the average score of JS is 0.8136, which is 1.28 points higher than UNet and 1.60 points better than UNet 3+. Furthermore, the average DC score of the CBAM+Ref-UNet 3+ is 0.8848 on skin cancer segmentation. Hence, the results show that our proposed CBAM+Ref-UNet 3+ is feasible and effective, and the segmentation performance was significantly improved.

#### 3.4.2. Breast Cancer Segmentation

In this experiment, the parameter settings in this implementation are the same as the skin cancer segmentation dataset. We used the ADAM optimization technique with a learning rate of 2 × 10^−4^, a number of iterations of 200, the data augmentation ratio of 0.5, and the loss function of dice coefficient loss.

**Figure 6:**
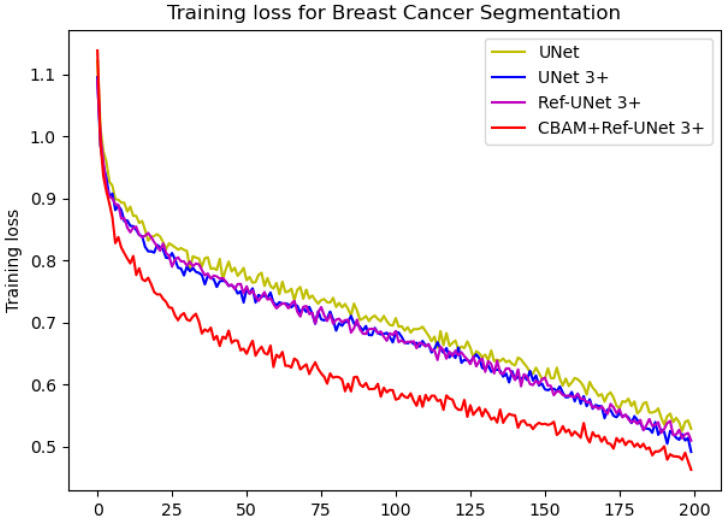
Comparison of training loss of different models for breast cancer segmentation.

**Figure 7:**
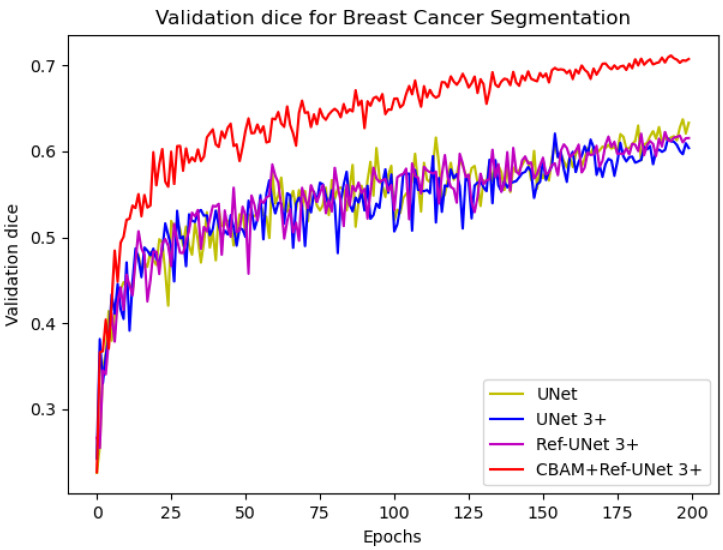
Comparison of validation dice of different models for breast cancer segmentation.

We can see from Figure 6 that the loss of the proposed model decreases faster and is able to obtain smaller losses. From the dice metric in Figure 7, we can show that the proposed model has the highest dice and climbs steadily with the number of iterations. Relatively speaking, CBAM+Ref-UNet 3+ can have the best segmentation performance.

Table 2 summarizes the results of different methods on this dataset. On F1-score, the average score of UNet is 0.6678, the average score of UNet 3+ is 0.6564, the average score of Ref-UNet 3+ is 0.6656, and the average score of CBAM+Ref-UNet 3+ is 0.6858. On JS, the score of CBAM+Ref-UNet 3+ is 0.5228, which is 2.16% and 3.38% higher than UNet and UNet 3+, respectively. On DC, the CBAM+Ref-UNet 3+ score is 0.7132, which are 6.28% and 7.98% higher than UNet and UNet 3+. Thereby, our proposed module provides better performance.

#### 3.4.3. Lung Segmentation

In this experiment, we used the ADAM optimization technique with a learning rate of 2 × 10^−4^. The number of iterations was 200, the data augmentation ratio was 0.5, and the loss function was dice coefficient loss. In addition, because the lung segmentation dataset is small and the images are not complex, we created a set of encoder layers with fewer convolutions: 8—>16—>32—>64—>128, and the decoder layers make corresponding adjustments according to different models.

**Figure 8:**
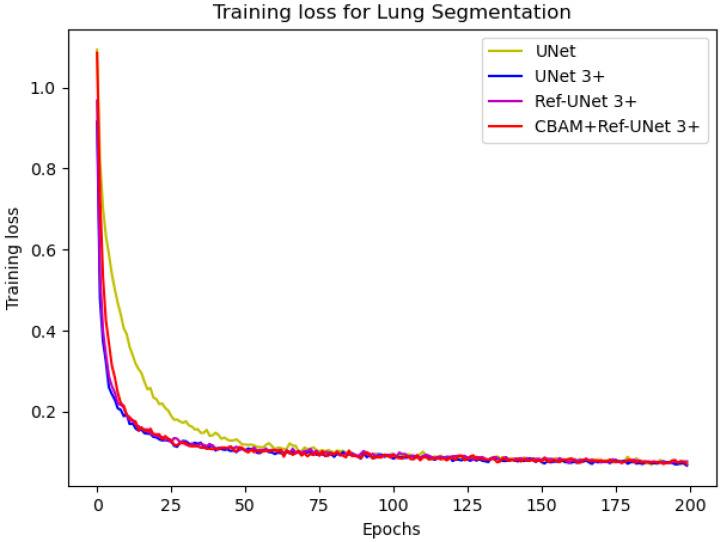
Training loss of the proposed models against UNet and UNet 3+.

**Figure 9:**
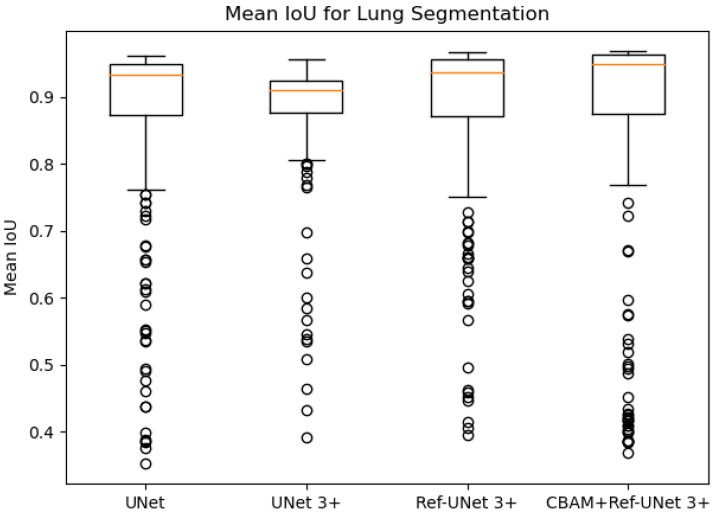
Mean IoU of the proposed models against UNet and UNet 3+.

Figure 8 and Figure 9 show the training loss and mean IoU when using the lung segmentation dataset. CBAM+Ref-UNet 3+ converges faster in training loss and provides the highest mean IoU score. Thus, the performance proves the validity of the proposed segmentation methods.

The comparison results are given in Table 3. It can be seen that CBAM+Ref-UNet 3+ achieves the highest average scores on multiple metrics. Moreover, CBAM+Ref-UNet 3+ can accomplish the best segmentation performance with fewer parameters.

### 3.5. Computation Time

Table 4 shows the number of parameters, floating point operations, training time and computation time of a test sample for each model using the skin cancer segmentation dataset as an example. Our improved models have fewer parameters and floating point operations. In addition, compared to UNet 3+, the proposed models have much shorter training times and computation times for a sample.

### 3.6. Visual Analysis

**Figure 10:**
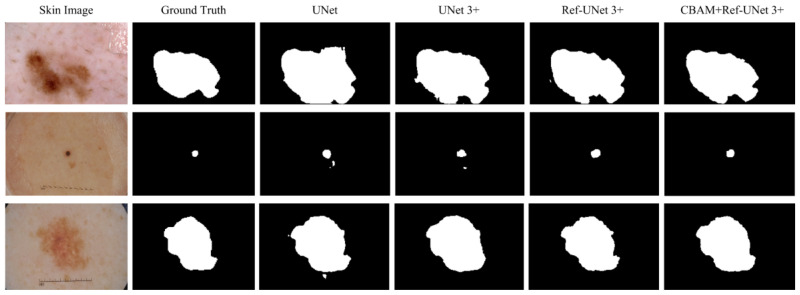
Visualization of skin cancer segmentation results. From left to right: original image, ground truth, segmentation result of Unet, segmentation result of Unet 3+, segmentation result of Ref-Unet 3+, and segmentation result of CBAM+Ref-Unet 3+.

**Figure 11:**
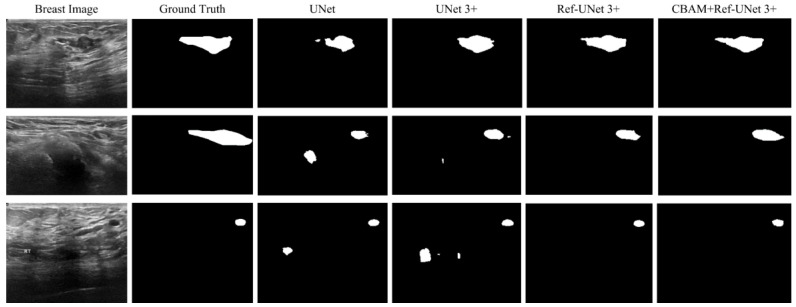
Visualization of breast cancer segmentation results. From left to right: original image, ground truth, segmentation result of Unet, segmentation result of Unet 3+, segmentation result of Ref-Unet 3+, and segmentation result of CBAM+Ref-Unet 3+.

**Figure 12:**
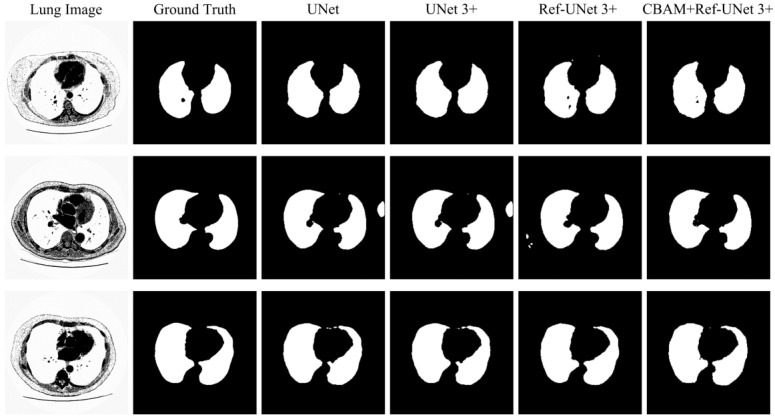
Visualization of lung segmentation results. From left to right: original image, ground truth, segmentation result of Unet, segmentation result of Unet 3+, segmentation result of Ref-Unet 3+, and segmentation result of CBAM+Ref-Unet 3+.

This section shows the partial visual segmentation results of the three datasets of skin, breast, and lung segmentation, as shown in Figure 10, Figure 11 and Figure 12, respectively. The segmentation results of each method are image binarized with a threshold of 0.5 [28]. Firstly, the presented methods are sharper in boundary segmentation in the skin image, comparable to GT pictures. Secondly, all methods’ segmentation results are not perfect in the breast image, but Ref-UNet 3+ and CBAM+Ref-Unet 3+ can accurately find the lesion. Lastly, in the lung image, the segmentation accuracy of each model was higher, but our models performed the best in handling details.

## 4. Discussion

The above results show that our proposed model is the best overall and is able to outperform other models in a variety of metrics. However, there are some drawbacks; for instance, the number of convolutions has a minimal impact on the performance on small data sets and medical images with simple structures. Therefore, the proposed model does not perform well enough, and further details need to be extracted and used to enhance the feature fusion.

In practical applications, it is more necessary to minimize network parameters and computation time than to increase the accuracy of current deep learning-based medical picture segmentation models. As a result, our first contribution is to propose an improved jump connection structure. Moreover, we added an attention mechanism to this model. The attention mechanism can selectively focus on the image regions of interest to obtain more detailed information, which can effectively improve the feature representation ability of the model. Finally, the various models were validated on three datasets.

First, we found that CBAM+Ref-UNet 3+ was optimal in all evaluation metrics on skin images with large sample sizes and distinct lesion boundaries. At the same time, the segmentation time used for the test set also indicated the best performance. Furthermore, we put up two network architectures using the small-sample breast dataset to see if our suggested models still have good segmentation accuracy with varying numbers of convolutions. The proposed two models outperform the comparative methods in various metrics, particularly PRE, JS, and DC. Finally, we use the lung cancer dataset to perform segmentation to see if the proposed models are valid. The lung is larger and more regular in shape than other organs. Hence the various models have limited potential to increase segmentation accuracy. However, our suggested model achieves the best segmentation performance with the fewest parameters because of the attention mechanism.

Although the research in this paper has achieved some results, the following areas need further exploration in the future: (1) We consider accelerating the convolution operation and optimizing the loss function to improve the performance of our models. (2) Deformable convolution is used to enhance the transformation modeling capability of CNNs [29,30,31,32,33], and it should be determined whether adding it to the model can enhance the feature extraction capability.

## 5. Conclusions

In this paper, we propose an improved model of UNet 3+ combined with CBAM. The goal of our studies was three-fold: Firstly, the proposed model achieves excellent segmentation performance with fewer parameters. Secondly, the proposed model enhances feature extraction’s ability to understand the image better while improving the accuracy and completeness of image segmentation. Lastly, the proposed model has better segmentation performance than UNet and UNet 3+.

## Figures and Tables

**Table 1:** Experimental results of proposed methods for skin cancer segmentation and comparison against other methods. The bold data denotes the best value.

Methods	Parameters	k-Fold	ACC	PRE	SE	F1-Score	JS	DC
UNet	32.92 M	1-fold	0.961	0.891	0.898	0.894	0.809	0.879
		2-fold	0.955	0.873	0.888	0.880	0.786	0.871
		3-fold	0.958	0.883	0.903	0.893	0.806	0.880
		4-fold	0.957	0.890	0.892	0.891	0.803	0.881
		5-fold	0.958	0.876	0.903	0.889	0.800	0.876
		Average	0.9578	0.8826	0.8968	0.8894	0.8008	0.8774
UNet 3+	25.71 M	1-fold	0.960	0.898	0.891	0.894	0.809	0.877
		2-fold	0.955	0.855	0.901	0.877	0.782	0.870
		3-fold	0.957	0.879	0.903	0.891	0.803	0.881
		4-fold	0.956	0.884	0.895	0.889	0.801	0.879
		5-fold	0.957	0.864	0.905	0.884	0.793	0.874
		Average	0.9570	0.8760	0.8990	0.8870	0.7976	0.8762
Ref-UNet 3+	21.00 M	1-fold	0.961	0.894	0.897	0.896	0.811	0.877
		2-fold	0.954	0.864	0.892	0.878	0.782	0.868
		3-fold	0.958	0.879	0.908	0.893	0.806	0.881
		4-fold	0.955	0.884	0.888	0.886	0.795	0.878
		5-fold	0.958	0.885	0.895	0.890	0.802	0.873
		Average	0.9572	0.8812	0.8960	0.8886	0.7992	0.8754
CBAM+Ref-UNet 3+	**21.02 M**	1-fold	0.963	0.900	0.901	0.900	0.819	0.882
		2-fold	0.958	0.886	0.892	0.889	0.800	0.879
		3-fold	0.961	0.893	0.911	0.901	0.821	0.889
		4-fold	0.959	0.892	0.901	0.896	0.812	0.885
		5-fold	0.961	0.898	0.900	0.899	0.816	0.889
		Average	**0.9604**	**0.8938**	**0.9010**	**0.8970**	**0.8136**	**0.8848**

**Table 2:** Experimental results of proposed methods for breast cancer segmentation and comparison against other methods. The bold data denotes the best one.

Methods	Parameters	k-Fold	ACC	PRE	SE	F1-Score	JS	DC
UNet	32.92 M	1-fold	0.942	0.600	0.687	0.641	0.470	0.636
		2-fold	0.935	0.670	0.673	0.672	0.506	0.658
		3-fold	0.933	0.716	0.640	0.676	0.511	0.660
		4-fold	0.952	0.669	0.683	0.676	0.511	0.657
		5-fold	0.934	0.649	0.701	0.674	0.508	0.641
		Average	0.9392	0.6608	0.6768	0.6678	0.5012	0.6504
UNet 3+	25.71 M	1-fold	0.944	0.564	0.723	0.634	0.464	0.624
		2-fold	0.937	0.644	0.698	0.670	0.504	0.650
		3-fold	0.934	0.712	0.648	0.679	0.514	0.650
		4-fold	0.935	0.729	0.554	0.629	0.459	0.613
		5-fold	0.930	0.672	0.668	0.670	0.504	0.630
		Average	0.9360	0.6642	0.6582	0.6564	0.4890	0.6334
Ref-UNet 3+	21.00 M	1-fold	0.942	0.572	0.704	0.631	0.461	0.624
		2-fold	0.938	0.695	0.689	0.692	0.529	0.672
		3-fold	0.937	0.682	0.679	0.681	0.516	0.670
		4-fold	0.948	0.650	0.660	0.655	0.487	0.652
		5-fold	0.931	0.644	0.695	0.669	0.494	0.6330
		Average	0.9392	0.6486	0.6854	0.6656	0.4974	0.6502
CBAM+Ref-UNet 3+	**21.02 M**	1-fold	0.942	0.625	0.678	0.650	0.482	0.701
		2-fold	0.945	0.700	0.734	0.717	0.558	0.736
		3-fold	0.933	0.731	0.624	0.673	0.507	0.718
		4-fold	0.961	0.705	0.760	0.731	0.577	0.739
		5-fold	0.927	0.665	0.651	0.658	0.490	0.672
		Average	**0.9416**	**0.6852**	**0.6894**	**0.6858**	**0.5228**	**0.7132**

**Table 3:** Experimental results of proposed methods for lung segmentation and comparison against other existing methods. The bold data denotes the best value.

Methods	Parameters	k-Fold	ACC	PRE	SE	F1-Score	JS	DC
UNet	0.60 M	1-fold	0.985	0.982	0.954	0.968	0.937	0.968
		2-fold	0.977	0.947	0.961	0.954	0.912	0.956
		3-fold	0.990	0.993	0.966	0.980	0.960	0.976
		4-fold	0.987	0.991	0.956	0.973	0.947	0.968
		5-fold	0.988	0.994	0.954	0.974	0.949	0.971
		Average	0.9854	0.9814	0.9582	0.9698	0.9410	0.9678
UNet 3+	0.40 M	1-fold	0.984	0.977	0.953	0.965	0.932	0.964
		2-fold	0.978	0.937	0.937	0.955	0.913	0.958
		3-fold	0.980	0.996	0.956	0.976	0.953	0.973
		4-fold	0.986	0.987	0.955	0.971	0.944	0.967
		5-fold	0.988	0.993	0.955	0.974	0.949	0.972
		Average	0.9832	0.9780	0.9512	0.9682	0.9382	0.9668
Ref-UNet 3+	0.33 M	1-fold	0.985	0.963	0.972	0.968	0.937	0.967
		2-fold	0.977	0.949	0.958	0.953	0.911	0.957
		3-fold	0.989	0.992	0.964	0.978	0.956	0.974
		4-fold	0.987	0.993	0.954	0.973	0.947	0.968
		5-fold	0.989	0.989	0.963	0.976	0.952	0.974
		Average	0.9854	0.9772	0.9622	0.9696	0.9406	0.9680
CBAM+Ref-UNet 3+	**0.33 M**	1-fold	0.988	0.975	0.974	0.975	0.950	0.975
		2-fold	0.979	0.949	0.964	0.956	0.917	0.959
		3-fold	0.990	0.995	0.964	0.979	0.959	0.977
		4-fold	0.988	0.990	0.961	0.975	0.951	0.971
		5-fold	0.991	0.994	0.967	0.980	0.961	0.979
		Average	**0.9872**	0.9806	**0.9660**	**0.9730**	**0.9476**	**0.9722**

**Table 4:** Comparison of models in terms of computation time.

Methods	Parameters (M)	GFLOPs	Training Time (h.)	Test Time (s)/Sample
UNet 3+	25.71	0.27	18	0.035
Ref-UNet 3+	21.00	0.22	6	0.015
CBAM+Ref-UNet 3+	21.02	0.22	7	0.020

## Data Availability

Publicly available datasets were analyzed in this study. These data can be found here: [https://www.kaggle.com] (accessed on 15 November 2022).
